# Liposomal Lipopolysaccharide Initiates TRIF-Dependent Signaling Pathway Independent of CD14

**DOI:** 10.1371/journal.pone.0060078

**Published:** 2013-04-02

**Authors:** Sachiko Watanabe, Yoshio Kumazawa, Joe Inoue

**Affiliations:** 1 Department of Biosciences, School of Science and Graduate School of Science, Kitasato University, Sagamihara, Kanagawa, Japan; 2 Department of Immunology and Cell Biology, Graduate School of Medicine, Kyoto University, Kyoto, Kyoto, Japan; National Institute of Infectious Diseases, Japan

## Abstract

Lipopolysaccharide (LPS) is recognized by CD14 with Toll-like receptor 4 (TLR4), and initiates 2 major pathways of TLR4 signaling, the MyD88-dependent and TRIF-dependent signaling pathways. The MyD88-dependent pathway induces inflammatory responses such as the production of TNF-α, IL-6, and IL-12 via the activation of NFκB and MAPK. The TRIF-dependent pathway induces the production of type-I IFN, and RANTES via the activation of IRF-3 and NFκB, and is also important for the induction of adaptive immune responses. CD14 plays a critical role in initiating the TRIF-dependent signaling pathway response to LPS, to support the internalization of LPS via endocytosis. Here, we clearly demonstrate that intracellular delivery of LPS by LPS-formulated liposomes (LPS-liposomes) initiate only TRIF-dependent signaling via clathrin-mediated endocytosis, independent of CD14. In fact, LPS-liposomes do not induce the production of TNF-α and IL-6 but induce RANTES production in peritoneal macrophages. Additionally, LPS-liposomes could induce adaptive immune responses effectively in CD14-deficient mice. Collectively, our results strongly suggest that LPS-liposomes are useful as a TRIF-dependent signaling-based immune adjuvant without inducing unnecessary inflammation.

## Introduction

Lipopolysaccharide (LPS) recognition has been well studied, and CD14 along with Toll-like receptor 4 (TLR4) forms the best-characterized LPS sensor [1]–[12]. CD14 was the first identified Pattern Recognition Receptor that binds directly to LPS [7], and is known to chaperone LPS to the TLR4 signaling pathway [13], [14]. TLR4 signaling is also well studied, and it is known that 2 major signaling pathways, the MyD88-dependent and TIR-domain-containing adapter-inducing interferon-β (TRIF)-dependent signaling pathways, are activated when TLR4 recognizes LPS [4]–[6], [15]. The MyD88-dependent pathway is activated at the plasma membrane and induces inflammatory responses such as the production of TNF-α, IL-6, and IL-12 via the activation of mitogen-activated protein kinase (MAPK) and nuclear factor kappa B (NFκB) in the early phase. On the other hand, the TRIF-dependent pathway is activated when LPS is taken into the endosome [16], [17]. In support of this, Kagan *et al.* used endocytosis inhibitors and showed that endocytosis of TLR4 with LPS initiates the TRIF-dependent pathway in early endosomes [18]. TRIF-dependent signaling induces the production of type-I interferon (IFN), which activates anti-viral responses, chemokines such as regulated upon activation, normal T cell expressed and secreted (RANTES; also known as CCL5), and to some extent, interleukin (IL)-6 via the activation of IFN regulatory factor (IRF)-3 and NFκB in the late phase [16], [19].

With regard to the relationship between the TRIF-dependent pathway and CD14, Zhengfan *et al.* showed, using Heedless mutation mice whose phenotype was positionally ascribed to a premature stop codon in *cd14,* that CD14 is required for the TRIF-dependent pathway [20]. Recently, it has been reported that CD14 controls the LPS-induced endocytosis of TLR4 through the tyrosine kinase Syk and its downstream effector phospholipase C (PLC) γ2 [21]. Taken together, the initiation of the TRIF-dependent signaling pathway by LPS requires endocytosis and CD14 supports this internalization of LPS. Additionally, CD14 also enhances the MyD88-dependent pathway activated with the plasma membrane response to LPS [10]. CD14-deficient mice showed resistance to endotoxin shock induced by LPS [9]. Therefore, CD14 plays a critical role in response to LPS.

As a candidate for a vaccine adjuvant, LPS can strongly induce and modulate adaptive immune responses, however, the LPS-initiated MyD88-dependent pathway induces the production of inflammatory cytokines such as TNF-α, IL-6, and IL-12, and this unnecessary inflammation sometimes causes septic shock with a cytokine storm [22]. On the other hand, the intracellular recognition of LPS initiates the TRIF-dependent pathway, which is important for the induction of adaptive immune responses [23], [24]. Hence, adjuvants that activate only the TRIF-dependent pathway are likely be safer; however, there is no tool to activate only the TRIF-dependent pathway response to LPS.

In this study, we newly prepared LPS-formulated liposomes (LPS-liposomes) to deliver the LPS directly to the endosome. We hypothesized that direct delivery of LPS to the endosome would activate the TRIF-dependent pathway and thereby form an effective immune adjuvant. As expected, LPS-liposomes were internalized via clathrin-mediated endocytosis and activated the TRIF-dependent pathway independent of CD14, but not the MyD88-dependent pathway. These results suggest that CD14 is required only for the uptake of LPS via endocytosis. Additionally, antigen-encapsulating LPS-liposomes could induce antigen-specific adaptive immune responses effectively in both wild-type and CD14-deficient mice. Taken together, LPS-liposomes can be useful as an immune adjuvant to induce protective immunity without inducing unnecessary inflammation.

## Materials and Methods

### Mice

Wild-type C57BL/10ScSn (WT), TLR4-deficient C57BL/10ScN (TLR4^−/−^) mice, and CD14-deficient C57BL/10 (CD14^−/−^) mice were obtained from the Max-Planck Institute for Immunobiology and Epigenetics (Freiburg, Germany). All mice were used at 8–12 weeks of age. All mice were housed in a specific pathogen-free environment at the Kitasato University School of Science in strict accordance with the Institutional Animal Care and Use Committee (IACUC) Guidelines. This study was carried out in strict accordance with the recommendations in the Guide for the Care and Use of Laboratory Animals of Kitasato University School of Science. The protocol was approved by the Committee on the Ethics of Animal Experiments of Kitasato University School of Science (Permit Number: SA1017). All efforts were made to minimize suffering.

### Reagents

Highly purified LPS from *S. abortus equi* was kindly provided by Dr. Chris Galanos (Max-Planck Institute for Immunobiology and Epigenetics). Alexa488-conjugated LPS, rhodamine-conjugated dextran (MW, 10,000 Da), and rhodamine-conjugated transferrin were purchased from Life Technologies Corporation (Tokyo, Japan). Brewer’s thioglycollate was purchased from BD Biosciences (Franklin Lakes, NJ). Anti-IRF3, anti-phospho-IRF3 (Ser396), anti-inhibitor of kappa B (IκB) α anti-phospho-IκBα (Ser32), anti- interleukin-1 receptor-associated kinase 1 (IRAK1), anti-glyceraldehyde phosphate dehydrogenase (GAPDH), anti-phospho-stress-activated protein kinase/c-Jun N-terminal kinase (SARK/JNK) (Thr183/Tyr185), anti-SARK/JNK, anti-phospho-p38 MAPK (Thr180/Tyr182), and anti-p38 MAPK antibodies for immunoblot analysis were purchased from Cell Signaling Technology (Danvers, MA). AP-conjugated goat anti-mouse IgG antibodies for the enzyme-linked immunosorbent assay (ELISA) were purchased from Invitrogen (Carlsbad, CA). 1,2-dioleoyl-3-trimethylammonium-propane (DOTAP) and 1,2-dipalmitoyl- sn-glycero-3-phosphoethanolamine-N-[methoxy (polyethylene glycol)-2000] (DPPE-PEG) were obtained from Avanti Polar Lipids (Birmingham, AL). Ovalbumin (OVA) and chlorpromazine hydrochloride (CPZ) were obtained from Sigma (St. Louis, MO).

### Preparation of Liposomes

DOTAP was dissolved in chloroform, vacuum-desiccated, and hydrated by vortexing with sterilized phosphate-buffered saline (PBS) for liposomes, and LPS or Alexa488-conjugated LPS (10–100 µg/mL) in sterilized PBS for LPS-liposomes, and diluted in PBS to obtain a final LPS concentration of 1 µg/mL. LPS (1 µg/mL), LPS-lipoosmes (LPS concentration of 1 µg/mL) and liposomes (same DOTAP concentration of LPS-liposomes) was diluted in the medium were used for the experiments. For the encapsulation of OVA, DOTAP and DPPE-PEG were dissolved in chloroform, vacuum-desiccated, and hydrated by vortexing with a mixture of LPS (1 mg/mL) and OVA (1 mg/mL) in PBS, and diluted in PBS to obtain a final concentration of 100 µg/mL. Following hydration, the dispersion was sonicated for 1 min in a bath sonicator (Bioruptor, Cosmo Bio, Tokyo, Japan).

### Cell Culture and Cytokine Determination

2 mL of 4% Brewer’s thioglycollate medium was injected intraperitoneally. Thioglycollate-elicited peritoneal macrophages were collected 4 days later using cold Hank’s balanced salt solution. Thioglycollate-elicited peritoneal macrophages from WT or CD14^−/−^ mice were cultured in 96-well plates (1×10^5^ cells per well) with LPS or LPS-liposomes (10 ng/mL) for 2 h (TNF-α) or 24 h (IL-6, and RANTES). In case of using endocytosis inhibitor, thioglycollate-elicited peritoneal macrophages from WT or CD14^−/−^ mice were cultured in 96-well plates (2×10^5^ cells per well) with LPS or LPS-liposomes (100 ng/mL) in the presence or absence of CPZ (0–50 µM). After 2 h (TNF-α), medium was changed and incubated for 24 h (IL-6, and RANTES). TNF-α, and IL-6 were measured by ELISA using the OptEIA mouse cytokine detection kit (BD Biosciences). RANTES was measured by ELISA using the Quantikine mouse RANTES ELISA Kit (R&D Systems, Minneapolis, MN). Thioglycollate-elicited peritoneal macrophages were incubated in RPMI-1640 medium supplemented with 10% fetal calf serum (FCS), 100 U/mL penicillin, 100 µg/mL streptomycin, 2 mM l-glutamine, and 5×10^–5^ M 2-mercaptoethanol.

### Flow Cytometry

Thioglycollate-elicited peritoneal macrophages from WT mice were cultured with Alexa488-conjugated LPS or LPS-liposomes (100 ng/mL) for 90 min. The cells were analyzed using a flow cytometer EPICS ELITE (Beckman Coulter, CA), and WinMDI for analysis software [25].

### Confocal Laser Scanning Microscopy

Thioglycollate-elicited peritoneal macrophages from WT mice were cultured with Alexa488-conjugated LPS or LPS-liposomes (100 ng/mL) for 90 min. In order to visualize clathrin-mediated endocytosis and macropinocytosis, the cells were incubated with rhodamine-conjugated transferrin (10 µg/mL) or rhodamine-conjugated dextran (100 µg/mL). The localization of fluorescence was determined by confocal laser scanning microscopy (Carl Zeiss, Jena, Germany) [26].

### Immunoblot Analysis

Thioglycollate-elicited peritoneal macrophages (1×10^6^ cells) from WT mice were cultured with LPS or LPS-liposomes (100 ng/mL) for 0–120 min. Cells were lysed with ice-cold RIPA lysis buffer containing protease inhibitors, and the extracts were subjected to immunoblot analysis. Immunoblot analysis was performed using anti-IRF3, anti-phospho-IRF3 (Ser396), anti-IκBα, anti-phospho-IκBα (Ser32), anti-IRAK1, anti-GAPDH, anti-pJNK (Thr183/Tyr185), anti-JNK, anti-pp38 MAPK (Thr180/Tyr182), and anti-p38 MAPK antibodies, visualized with HRP conjugate substrate system or an enhanced chemiluminescence detection system. Band intensity was quantified with Image J 1.45.

### OVA-specific Antibody Determination

WT or CD14^−/−^ mice were immunized with LPS plus OVA, or LPS-OVA-liposomes (10 µg each per mouse, intravenous) and boosted after 2 weeks. After a further 2 weeks, sera were harvested and poured into an OVA-coated ELISA-plate, and incubated overnight. Following washes with PBS containing 0.05% Tween-20, the wells were treated with AP-conjugated-anti-mouse IgG for 2 h. Following washes, an enzyme reaction was performed using *p*-nitrophenyl phosphate as the substrate. The absorbance at 405 nm (with a reference at 540 nm) was measured using a Bio-Rad Model 550 Microplate Reader (Bio-Rad, Hercules, CA), as described previously [27]–[30].

### Statistical Analysis

The paired Student’s *t* test was used to compare paired groups. *P* values <0.05 were considered significant.

## Results

### Internalization of LPS by Liposomes via Clathrin-mediated Endocytosis

**Figure 1:**
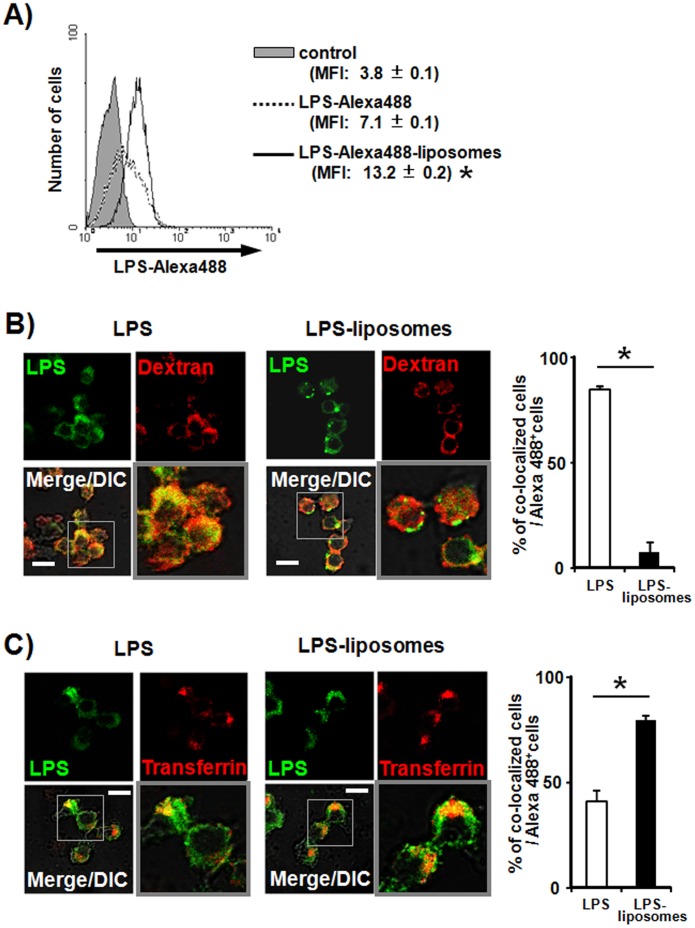
Internalization of lipopolysaccharide (LPS) by liposomes *via* clathrin-mediated endocytosis. (A) Thioglycollate-elicited peritoneal macrophages from wild-type (WT) mice were cultured with PBS or Alexa488-conjugated LPS (100 ng/mL) or Alexa488-conjugated LPS-liposomes (100 ng/mL) for 90 min. LPS uptake by macrophages was analyzed by flow cytometry. PBS treated macrophages were overlaid as control (gray histograms). Mean fluorescence intensity (MFI) are average of three independent experiments. The values represent means ± S.E.M * *P*<0.05. (B) and (C) Thioglycollate-elicited peritoneal macrophages from WT mice were cultured with Alexa488-conjugated LPS or LPS-liposomes (100 ng/mL) for 90 min with or without rhodamine-conjugated transferrin (10 µg/mL) or rhodamine-conjugated dextran (100 µg/mL). The localization of fluorescence was determined by confocal laser scanning microscopy. Scale bar: 10 µm. Histogram of the values for co-localized cells (cells with yellow signals) as a percent of the total Alexa488^+^ cells. 100 cells (Alexa488^+^ cells) were counted for each set and calculated on three separate experiments. The values represent means ± S.E.M * *P*<0.05.

First, we examined the uptake of LPS and LPS-liposomes by macrophages using Alexa488-conjugated LPS. The uptake of LPS-liposomes by macrophages was more efficient than that of LPS (Fig. 1A). It is reported that CD14-dependent internalization of LPS depended on 2 major endocytotic pathways, the macropinocytic pathway and the clathrin-mediated pathway [31], [32]. Therefore, we next examined the route of internalization of LPS and LPS-liposomes using fluorescence-labeled dextran to trace the macropinocytic pathway, and fluorescence-labeled transferrin to trace the clathrin-mediated pathway [33]. As shown in Fig. 1B, Alexa488-labeled LPS partially co-localized with rhodamine-labeled dextran; however, the localization of LPS-liposomes did not occur or very few. The percentage of co-localized cells was also significantly decreased in macrophages treated with LPS-liposomes. In case of the clathrin-mediated pathway, the fluorescence of both LPS and LPS-liposomes overlapped with rhodamine-labeled transferrin, and the percentage of co-localized cells was significantly increased in macrophages treated with LPS-liposomes (Fig. 1C). These results suggest that internalization of LPS was mediated by both the macropinocytic pathway and the clathrin-mediated pathway, and that LPS-liposomes were efficiently internalized mainly via the clathrin-mediated pathway.

### LPS-liposomes Induce the TRIF-dependent Signaling Pathway but not the MyD88-dependent Signaling Pathway

**Figure 2:**
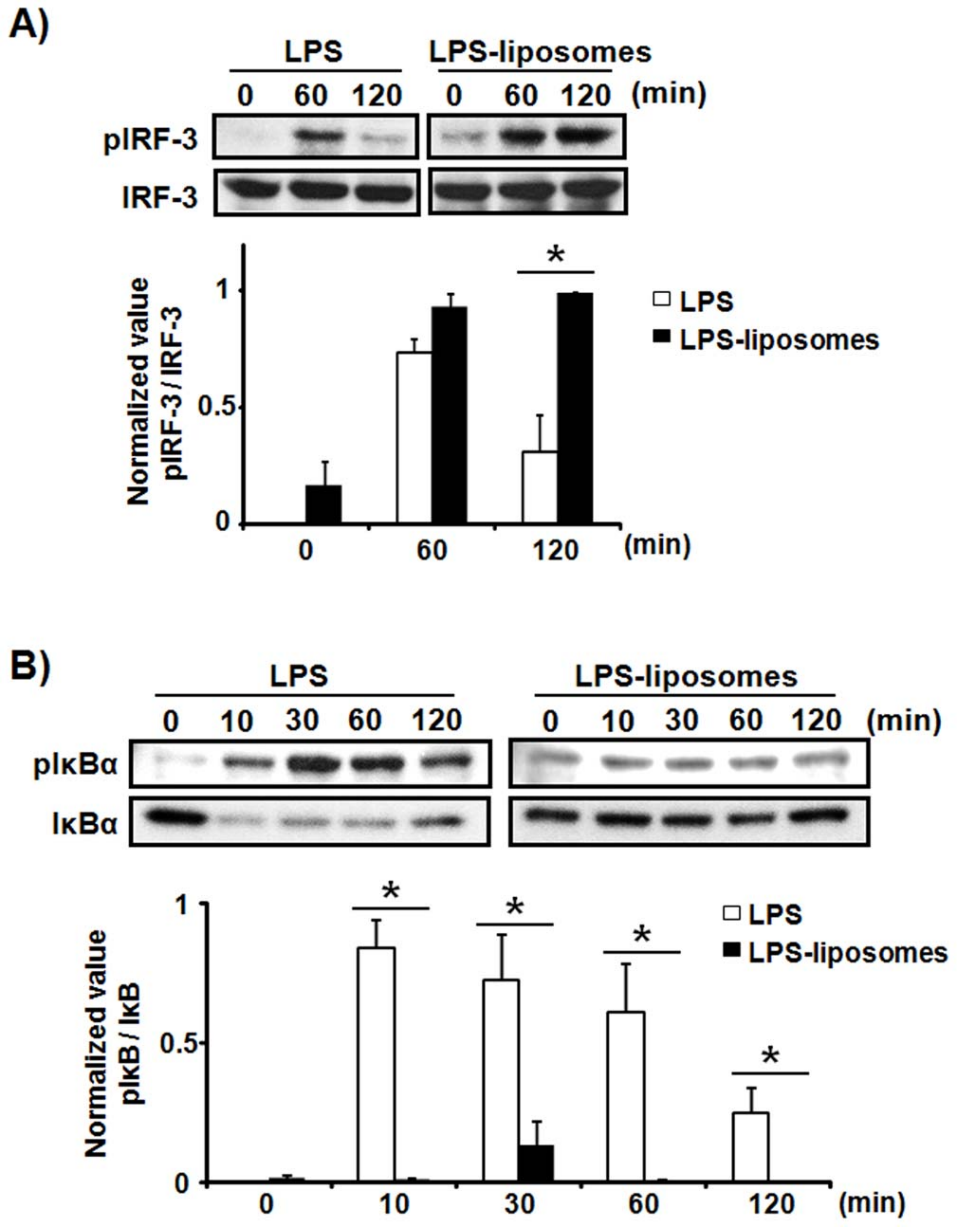
LPS-liposomes induce the TRIF-dependent signaling pathway but not the MyD88-dependent signaling pathway. Thioglycollate-elicited peritoneal macrophages (1×10^6^ cells) from WT mice were stimulated with LPS (100 ng/mL) or LPS-liposomes (100 ng/mL) for 0–120 min. The cells were then lysed and the extracts immunoblotted with anti-IRF3 (A), anti-IκBα antibodies (B), or anti-IRAK and anti-MAPK antibodies (C). Data are average of three independent experiments and band intensity was quantified with Image J 1.45. The values represent means ± S.E.M * *P*<0.05.

It has been reported that the activation of NFκB in the early phase is induced by MyD88-dependent signaling, and the activation of IRF-3 and NFκB in the late phase is induced by TRIF-dependent signaling [17]. Additionally, the TRIF-dependent pathway requires the endocytosis of TLR4 with LPS [18]. Therefore, we next examined the activation of NFκB and IRF-3 by LPS and LPS-liposomes. IRF-3 activation was detected by its phosphorylation, and NFκB activation was detected by the degradation and phosphorylation of IκBα, which inhibits NFκB activation. As shown in Figure 2A, the phosphorylation of IRF-3 was induced by both LPS and LPS-liposomes. Furthermore, the phosphorylation of IRF-3 was significantly enhanced and long-lasting upon LPS-liposomes treatment compared with LPS. In the case of IκBα, degradation and phosphorylation were only detected in response to LPS (Fig. 2B). We also examined the activation of IRAK1, an important molecule in the MyD88-dependent pathway, and the activation of MAPK. IRAK1 activation was measured by its degradation, and it was significantly detected in response to LPS compared with LPS-liposomes (Fig. 2C). The activation of MAPK was also induced by LPS; however, it was very weak in response to LPS-liposomes (Fig. 2C). These results strongly suggest that LPS-liposomes activate only the TRIF-dependent signaling pathway.

### LPS-liposomes Induce RANTES Production but not TNF-α and IL-6 Production in Macrophages from WT and CD14^−/−^ Mice

**Figure 3:**
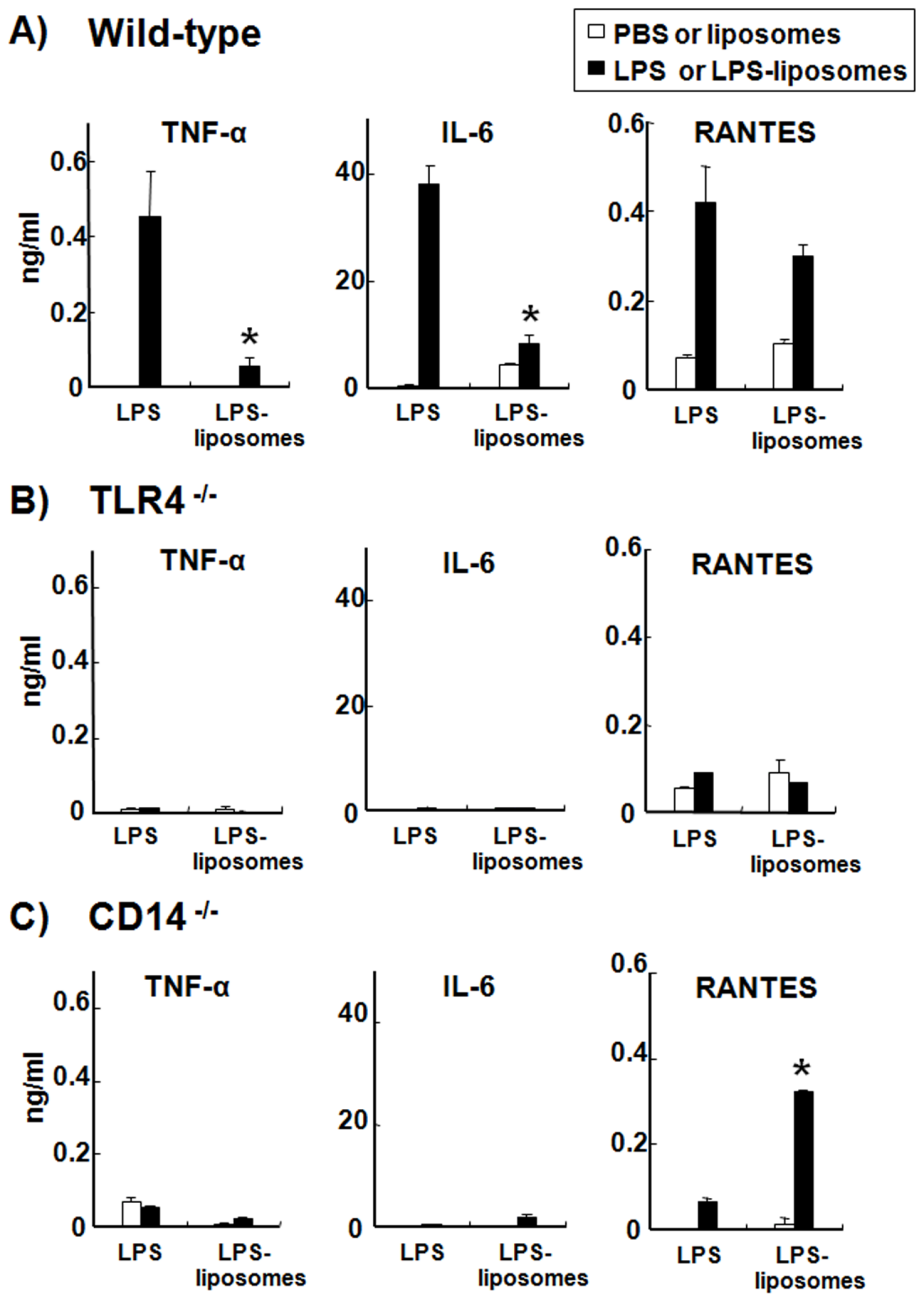
LPS-liposomes induce RANTES production but not TNF-α and IL-6 production in macrophages from WT and CD14^−/−^ mice. Thioglycollate-elicited peritoneal macrophages (1×10^5^ cells) from WT (A), TLR4^−/−^ (B), or CD14^−/−^ mice (C) were cultured with LPS or LPS-liposomes (10 ng/mL) for 2 h (TNF-α) or 24 h (IL-6, and RANTES). Cytokine levels were determined by ELISA. PBS was control for LPS and liposomes was control for LPS-liposomes (Open columns). Data are average of three independent experiments. The values represent means ± S.E.M * *P*<0.05 (LPS *vs.* LPS-liposomes).

Next, we examined the production of inflammatory cytokines and chemokines, which are induced via the MyD88-dependent and TRIF-dependent pathways. MyD88-dependent cytokines such as TNF-α and IL-6 were highly produced by WT macrophages in response to LPS (Fig. 3A). By contrast, LPS-liposomes did not induce these inflammatory cytokines. The production of RANTES, a TRIF-dependent chemokine, was induced by both LPS and LPS-liposomes (Fig. 3A). The production of inflammatory cytokines and chemokines were completely abolished in TLR4^−/−^ macrophages in response to both LPS and LPS-liposomes (Fig. 3B). Surprisingly, LPS-liposomes could induce RANTES production from CD14^−/−^ macrophages, although CD14^−/−^ macrophages did not exhibit any cytokine and chemokine response to LPS (Fig. 3C). Furthermore, LPS-liposomes could induce the phosphorylation of IRF-3 significantly compared with LPS (Fig. S1). These results suggest that LPS-liposomes activate the TLR4-mediated TRIF-dependent pathway independent of CD14.

### Clathrin-mediated Endocytosis of LPS Induces RANTES Production in Macrophages from WT and CD14^−/−^ Mice

**Figure 4:**
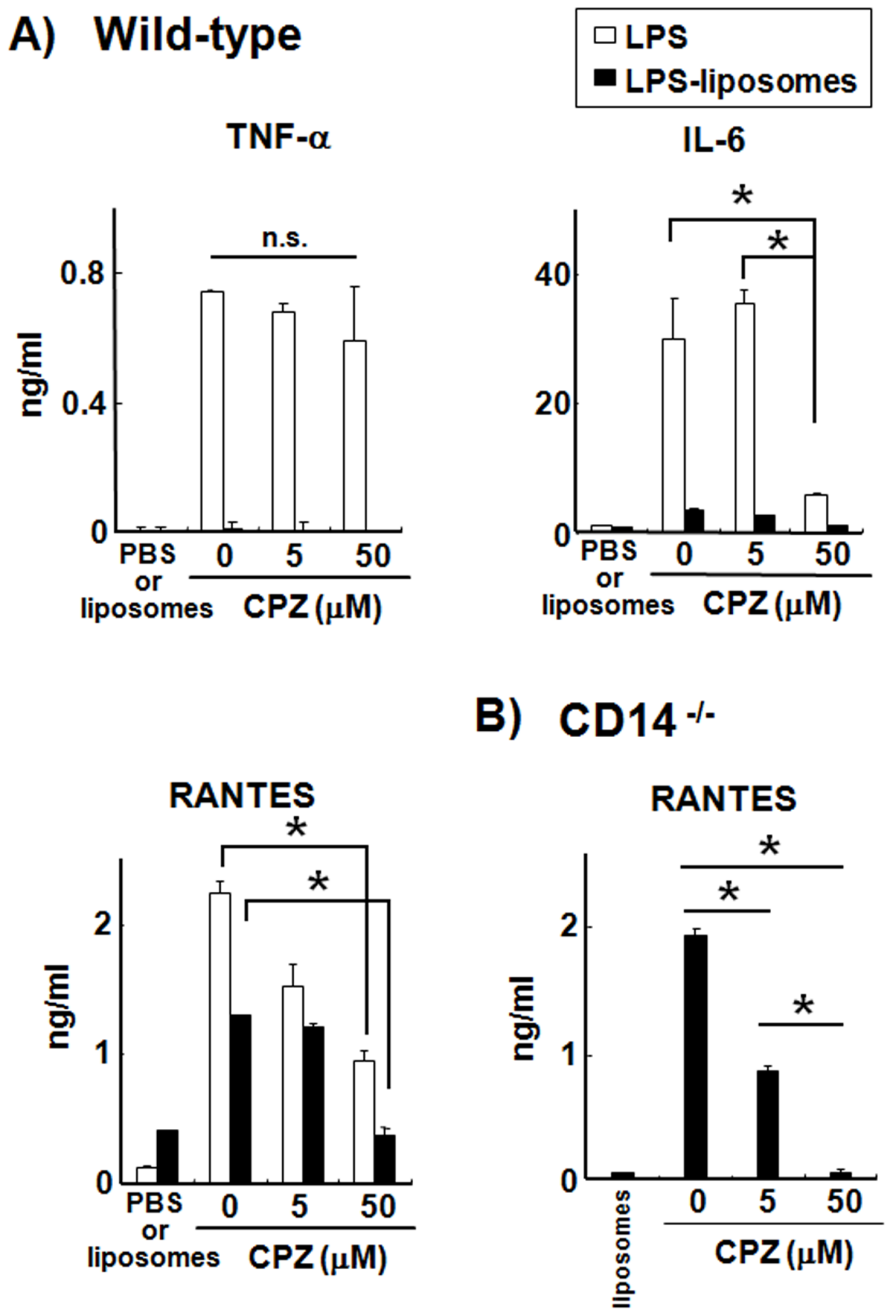
Clathrin-mediated endocytosis of LPS induces RANTES production in macrophages from WT and CD14^−/−^ mice. Thioglycollate-elicited peritoneal macrophages (2×10^5^ cells) from WT (A) or CD14^−/−^ mice (B) were cultured with LPS or LPS-liposomes (100 ng/mL) in the presence or absence of CPZ (0–50 µM). After 2 h (TNF-α), medium was changed and incubated for 24 h (IL-6, and RANTES). Cytokine levels were determined by ELISA. Data are average of three independent experiments. The values represent means ± S.E.M * *P*<0.05.

The TRIF-dependent pathway is initiated by the endocytosis of TLR4 with LPS [18]. Therefore, we examined the influence of an endocytosis inhibitor, CPZ, which inhibits the clathrin-mediated endocytosis pathway by which LPS and LPS-liposomes were internalized. As shown in Fig. 4A, LPS-induced TNF-αproduction was not affected by CPZ at all. The production of IL-6 and RANTES was affected partially. By contrast, LPS-liposome-induced RANTES production was completely inhibited by CPZ in WT and CD14^−/−^ macrophages (Fig. 4A,B). Moreover, the difference was not observed in cell viability at least 50 µM of CPZ (Fig. S2). These results strongly suggest that clathrin-mediated endocytosis is required to initiate the TRIF-dependent pathway for both LPS and LPS-liposomes, and that CD14 supports this clathrin-mediated endocytosis in the case of LPS.

### LPS-liposomes Enhances Antigen-specific IgG Production Independent of CD14

**Figure 5:**
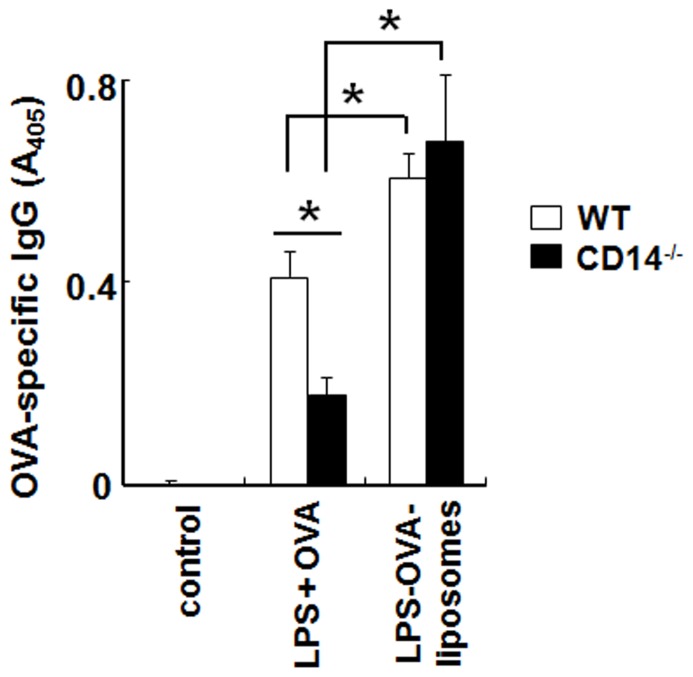
LPS-liposomes enhance antigen-specific IgG production independent of CD14. WT and CD14^−/−^ mice were immunized with LPS plus OVA or LPS-OVA-liposomes (10 µg each per mouse, intravenous), and boosted after 2 weeks. After a further 2 weeks, sera were harvested and OVA-specific IgG levels were determined by ELISA. Data are averages of two independent experiments. *n* = 5−8 animals per group. The values represent means ± S.E.M * *P*<0.05.

The activation of the TRIF-dependent pathway by the intracellular recognition of LPS is important for the induction of adaptive immune responses [23], [24], [34]. Therefore, we prepared ovalbumin (OVA)-encapsulating LPS-liposomes (LPS-OVA-liposomes) to examine the induction of antigen-specific adaptive immune responses in WT and CD14^−/−^ mice. Mice were immunized with LPS plus OVA or LPS-OVA-liposomes and boosted after 2 weeks. After a further 2 weeks, sera were harvested and OVA-specific antibodies were examined by ELISA. As shown in Figure 5, the production of OVA-specific IgG was significantly enhanced by LPS-OVA-liposomes in both WT and CD14^−/−^ mice compared with LPS plus OVA. In LPS plus OVA immunization, the antibody response was decreased significantly in CD14^−/−^ mice. These results suggest that CD14 is important for inducing the adaptive immune response by LPS, and LPS-OVA-liposomes effectively induce antigen-specific immune responses via the activation of the TRIF-dependent pathway independent of CD14 *in vivo*.

## Discussion

We newly prepared LPS-formulated liposomes to deliver the LPS directly to the endosome and showed that only the TRIF-dependent pathway was activated, independent of CD14. CD14 plays a critical role in LPS recognition and chaperones LPS to the TLR4 signaling pathway; therefore CD14^−/−^ mice showed resistance to endotoxin shock induced by LPS [9]. On the other hand, it has been reported that LPS from rough colonies (R-LPS), which lacks an *O*-polysaccharide chain of LPS, and Lipid A can induce inflammatory cytokines such as TNF-α in the absence of CD14 [20], [35]. However, TRIF-dependent cytokines such as I-IFN are not produced in the absence of CD14 even with R-LPS and Lipid A stimulation [20]. These studies indicate that CD14 is not indispensable for the MyD88-dependent pathway but required for the TRIF-dependent pathway. It has also been reported that CD14 controls the LPS-induced endocytosis of TLR4 to initiate the TRIF-dependent signaling pathway [21]. From our findings, LPS-liposomes could induce RANTES production in CD14^−/−^ macrophages and also induce effective immune responses in CD14^−/−^ mice (Figs. 3C, 5 and Fig. S1). Additionally, clathrin-mediated endocytosis of LPS was critically important for initiating the TRIF-dependent signaling pathway (Fig. 4). These results suggest that CD14 is not indispensable for TLR4 signaling, but endocytosis is essential.

LPS-liposomes induce the TRIF-dependent signaling pathway efficiently, but not the MyD88-dependent signaling pathway (Fig. 2, 3). This is because LPS is highly concentrated in the liposomes and escape recognition by LPS sensors at the plasma membrane. Liposomes are also useful as drug carriers to efficiently deliver drugs, DNA, and immune stimulatory molecules into the cell via endocytosis [36]–[39]. It does not leak in general, LPS-liposomes deliver LPS into the cell more efficiently (Fig. 1). This uptake of highly concentrated LPS initiates the TRIF-dependent signaling pathway in endosomes. It has been reported that liposomes could spatiotemporally regulate CpG oligodeoxynucleotide-induced TLR9 signaling [40]. CpG-liposome complexes are retained for long periods in endosomal vesicles and induce robust IFN production *via* IRF-7 activation. Our LPS-liposomes is also manipulated for endosomal retention and regulates the intracellular recognition of LPS and TLR4 signaling, especially the TRIF-dependent signaling pathway. In fact, we detected the potentiation and extension of IRF-3 activation by LPS-liposomes (Fig. 2A), and it is predicted that this activation depends on the endosomal retention of LPS regulated by liposomes.

Recently, TLR ligands have been considered as candidates for immune adjuvants, given their ability to induce strong immune responses. However, these strong immune responses also induce unnecessary inflammation, which is sometimes fatal. Thus, it is very difficult to develop effective and safe immune adjuvants. It has recently been reported that TRIF-biased TLR4 agonists can be used as vaccine adjuvants with low toxicity [24]. Immunization with these agonists tends to activate the TRIF-dependent pathway rather than the MyD88-dependent pathway; therefore, the induction of unnecessary inflammation is minimal. It is also reported that the expression of MHC-class II and co-stimulatory molecules such as CD40, CD80, and CD86, which are important for the stimulation of naïve T cells to differentiate into effector T helper cells, could be induced by LPS in MyD88^−/−^ antigen-presenting cells, suggesting that the TRIF-dependent pathway plays a critical role in the induction of adaptive immunity [34]. These studies also support our observations that LPS-liposomes could serve as safe and effective immune adjuvants. Furthermore, the LPS-liposome can encapsulate antigen and induce antibody responses more effectively than LPS plus antigen (Fig. 5).

In conclusion, the LPS-liposome is useful as a TRIF-dependent signaling-based immune adjuvant that effectively induces adaptive immune responses without inducing unnecessary inflammation. The LPS-liposome is the first tool to induce only the TRIF-dependent pathway together with allowing antigen encapsulation, and may be useful as an immune adjuvant to induce protective immunity.

## Supporting Information

Figure S1 **LPS-liposomes induce the activation of IRF-3 in macrophages from CD14^−/−^ mice.** Thioglycollate-elicited peritoneal macrophages (1×10^6^ cells) from CD14^−/−^ mice were stimulated with LPS (100 ng/mL) or LPS-liposomes (100 ng/mL) for 0–120 min. The cells were then lysed and the extracts immunoblotted with anti-IRF3 and anti-pIRF3 antibodies. Data are average of three independent experiments and band intensity was quantified with Image J 1.45. The values represent means ± S.E.M * *P*<0.05.
(TIF)

Figure S2 **Cell viability of macrophages treated with CPZ.** Thioglycollate-elicited peritoneal macrophages (2×10^5^ cells) from WT mice were cultured in the presence or absence of CPZ (0–100 µM). After 2 h, medium was changed and incubated for 24 h. Cell viability was examined by Cell-titer Glo (Promega, Japan). Data are average of three independent experiments. The values represent means ± S.E.M * *P*<0.05 (0 µM vs 100 µM ).
(TIF)

Text S1 **Materials and Methods.**
(DOCX)
