# Supplementary figures and images for: Liposomal Lipopolysaccharide Initiates TRIF-Dependent Signaling Pathway Independent of CD14
Source: PLoS One. 2013 Apr 2;8(4):e60078. doi: 10.1371/journal.pone.0060078 (PMC3615118; doi:10.1371/journal.pone.0060078)

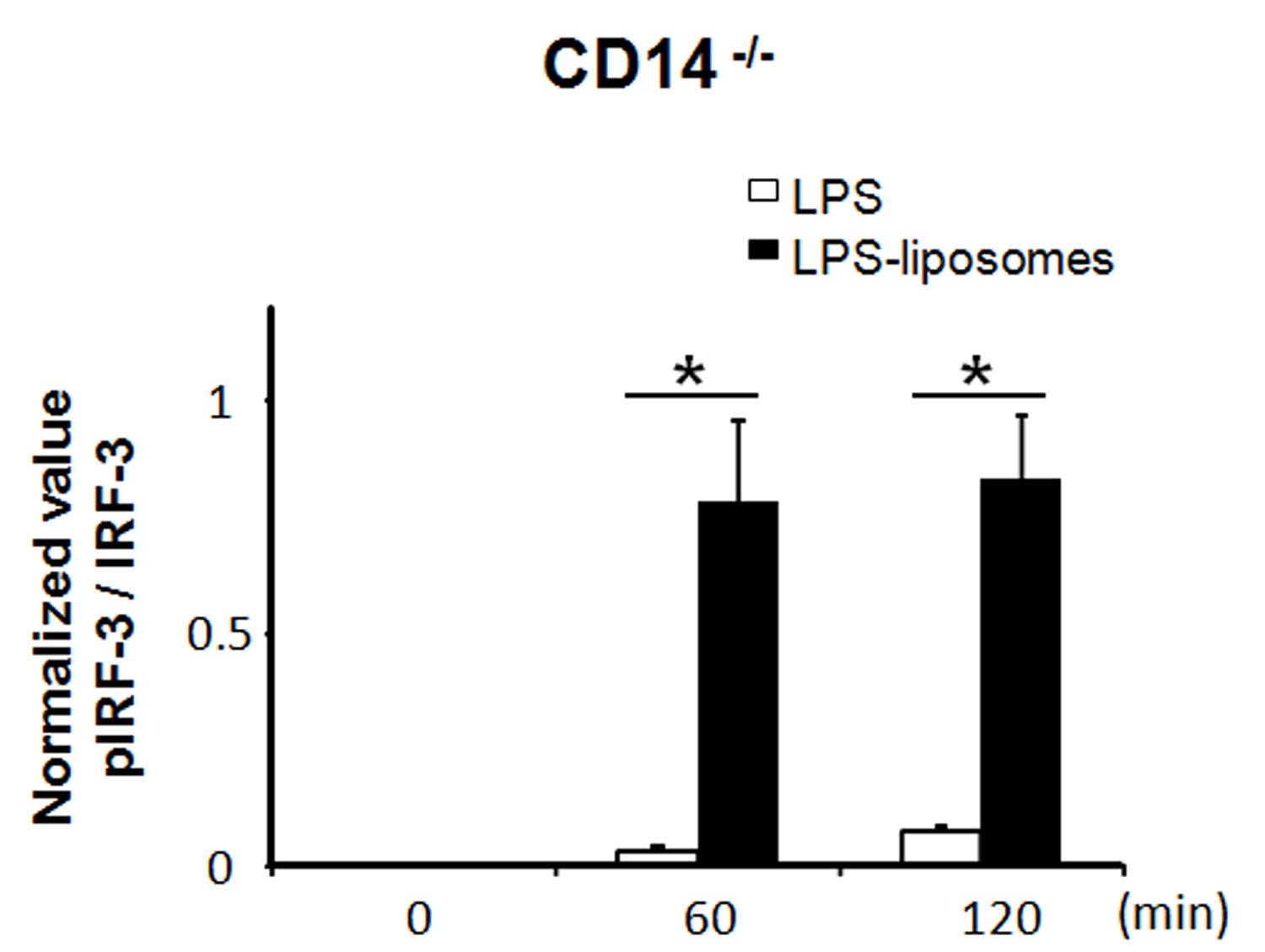

Supplement: Figure S1 — LPS-liposomes induce the activation of IRF-3 in macrophages from CD14−/− mice. Thioglycollate-elicited peritoneal macrophages (1×10^6^ cells) from CD14−/− mice were stimulated with LPS (100 ng/mL) or LPS-liposomes (100 ng/mL) for 0–120 min. The cells were then lysed and the extracts immunoblotted with anti-IRF3 and anti-pIRF3 antibodies. Data are average of three independent experiments and band intensity was quantified with Image J 1.45. The values represent means ± S.E.M * P<0.05. (TIF) [file pone.0060078.s001.tif]

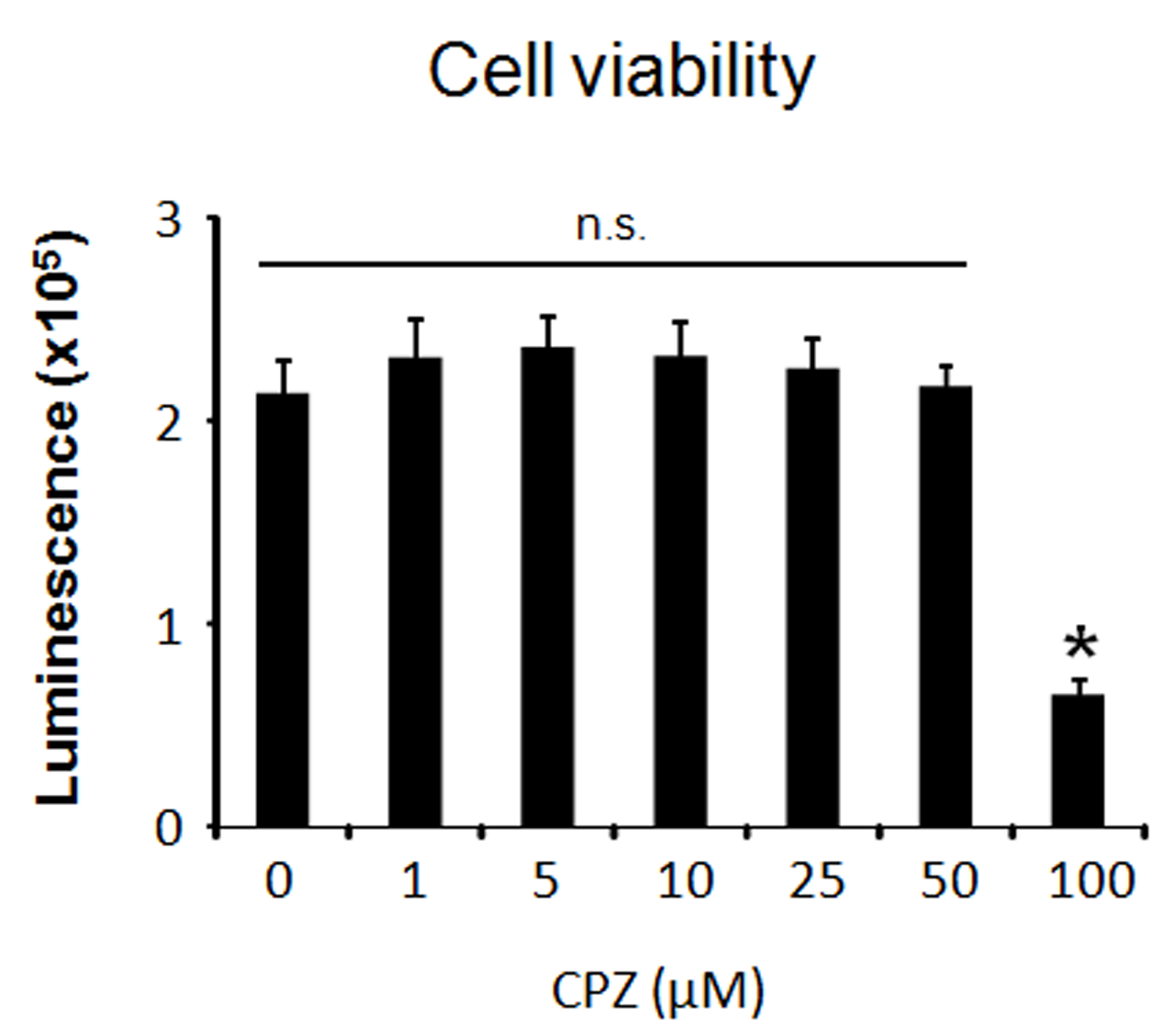

Supplement: Figure S2 — Cell viability of macrophages treated with CPZ. Thioglycollate-elicited peritoneal macrophages (2×10^5^ cells) from WT mice were cultured in the presence or absence of CPZ (0–100 µM). After 2 h, medium was changed and incubated for 24 h. Cell viability was examined by Cell-titer Glo (Promega, Japan). Data are average of three independent experiments. The values represent means ± S.E.M * P<0.05 (0 µM vs 100 µM ). (TIF) [file pone.0060078.s002.tif]
