# Supplementary material for: Liposomal Lipopolysaccharide Initiates TRIF-Dependent Signaling Pathway Independent of CD14
Source: PLoS One. 2013 Apr 2;8(4):e60078. doi: 10.1371/journal.pone.0060078 (PMC3615118; doi:10.1371/journal.pone.0060078)
Supplement: Text S1 — Materials and Methods. (DOCX) [file pone.0060078.s003.docx]

**Supporting Information**

**Materials and Methods**

***Immunoblot analysis***

Thioglycollate-elicited peritoneal macrophages (1 × 10^6^ cells) from CD14^-/-^ mice were cultured with LPS or LPS-liposomes (100 ng/mL) for 0–120 min. Cells were lysed with ice-cold RIPA lysis buffer containing protease inhibitors, and the extracts were subjected to immunoblot analysis. Immunoblot analysis was performed using anti-IRF3 and anti-phospho-IRF3 (Ser396), visualized with HRP conjugate substrate system. Band intensity was quantified with Image J 1.45.

***Cell viability assay***

Thioglycollate-elicited peritoneal macrophages from WT mice were cultured in 96-well plates (2 × 10^5^ cells per well) in the presence or absence of CPZ (0–100 μM). After 2 h, medium was changed and incubated for 24 h. Cell viability was examined by Cell-titer Glo (Promega, Japan).

**Supporting Figure legends**

**Fig. S1 LPS-liposomes induce the activation of IRF-3 in macrophages from CD14^-/-^ mice.** Thioglycollate-elicited peritoneal macrophages (1 × 10^6^ cells) from CD14^-/-^ mice were stimulated with LPS (100 ng/mL) or LPS-liposomes (100 ng/mL) for 0–120 min. The cells were then lysed and the extracts immunoblotted with anti-IRF3 and anti-pIRF3 antibodies. Data are average of three independent experiments and band intensity was quantified with Image J 1.45. The values represent means ± S.E.M *****P<0.05.

**Fig. S2 Cell viability of macrophages treated with CPZ.** Thioglycollate-elicited peritoneal macrophages (2 × 10^5^ cells) from WT mice were cultured in the presence or absence of CPZ (0–100 μM). After 2 h, medium was changed and incubated for 24 h. Cell viability was examined by Cell-titer Glo (Promega, Japan). Data are average of three independent experiments. The values represent means ± S.E.M *****P<0.05 (0 μM vs 100 μM ).
